# First-Trimester Morphological Evaluation of Fetuses and Medical Law Implications

**DOI:** 10.3390/diagnostics15101277

**Published:** 2025-05-18

**Authors:** Răzvan Grigoraș Căpitănescu, Marius Cristian Marinaș, Larisa Pătru, Dragoș George Popa, Elena Cristina Andrei, Aura Iuliana Popa, Gabriel Florin Răzvan Mogoș, Nicolae Dragoș Mărgăritescu, Ciprian Laurențiu Pătru

**Affiliations:** 1 Department of Obstetrics and Gynecology, University of Medicine and Pharmacy of Craiova, 200349 Craiova, Romania; razvan.capitanescu@gmail.com (R.G.C.); patru.ciprianl@gmail.com (C.L.P.); 2 Department of Human Anatomy, University of Medicine and Pharmacy of Craiova, 200349 Craiova, Romania; 3 Department 9, University of Medicine and Pharmacy of Craiova, 200349 Craiova, Romania; elena.andrei@umfcv.ro; 4 Department of Plastic Surgery, University of Medicine and Pharmacy of Craiova, 200349 Craiova, Romania; popa_dragos2000@yahoo.com; 5 Doctoral School, University of Medicine and Pharmacy of Craiova, 200349 Craiova, Romania; aura.nedianu@yahoo.com; 6 Department of General Surgery, University of Medicine and Pharmacy of Craiova, 200349 Craiova, Romania; gabrielmogos@yahoo.com (G.F.R.M.); dmargaritescu@yahoo.com (N.D.M.)

**Keywords:** fetal anomalies, first trimester, ultrasound

## Abstract

**Background/Objectives:** Over the years, the potential of the first-trimester (FT) ultrasound in the detection of fetal structural defects has increased. The main objectives of the first-trimester fetal screening evaluation are the detection of major structural anomalies and the diagnosis of additional sonographic markers for chromosomal disorders. When a fetal anomaly is diagnosed, patients have the right to be informed about the risks, necessary interventions, or alternatives. Depending on the severity of the anomalies and the pregnancy period, the legality of the pregnancy termination was evaluated. The aim of this study was to assess the impact of the first-trimester morphological screening of the fetus using an ultrasound protocol according to the latest international protocols (the ISUOG protocol). **Methods:** Between 1 January 2024 and 31 December 2024, 854 pregnancies with gestational ages between 11 weeks and 13 weeks + 6 days were morphologically evaluated during the nuchal scan in the Obstetrics and Gynecology Department of the Emergency County Hospital from Craiova. Both transabdominal and transvaginal ultrasound in 2D and in a color Doppler mode were used in the scanning technique. The ultrasound findings were correlated with the genetic testing results and pregnancy outcome. The medical law implications were related to the cases where the ultrasound was performed at about 13 weeks of gestation, and the screening genetic results showed an increased pregnancy risk, which arose during the FT. In these cases, we performed amniocentesis at about 16–17 weeks of gestation, and especially, the Non-Invasive Prenatal Testing (NIPT)-positive cases were confirmed by karyotyping. Still, at this gestational age of diagnosis, the Romanian law would not allow abortions. **Results**: By using this extended FT ultrasound protocol, we detected 58 cases with fetal structural anomalies. Eighteen cases were also associated with genetic syndromes after performing chorionic villous sampling (CVS). Three cases detected with minor structural anomalies (two cases with club foot and one case with a cleft upper lip) were lost to follow-up. **Conclusions:** Fetal morphological ultrasound evaluation is feasible in the late first trimester. By using an extended ultrasound protocol, we can detect most of the fetal structural anomalies and contribute to better medical counseling and improve pregnancy outcomes.

## 1. Introduction

The main objectives of the first-trimester fetal screening evaluation are the detection of the major structural anomalies and the diagnosis of additional sonographic markers for chromosomal disorders [1,2,3].

The measurement of the nuchal translucency (NT) for Down’s syndrome has become an important marker for genetic screening [4,5]. An ultrasound screening for fetal structural anomalies in the FT decreases the diagnostic time, reduces the late-term termination of pregnancy, and provides appropriate counseling for the couples [6,7]. However, the ability of the FT ultrasound to detect different fetal anomalies is variable, from never detectable to sometimes detectable to always detectable [8].

The use of cell-free DNA testing has decreased the role of NT in the first-trimester genetic screening [9].

The literature shows that to improve fetal anomaly detection rates in the FT, it is necessary to use a morphological protocol [10,11]. Differences between the study populations and screening protocols have resulted in varying results regarding the performance of the FT ultrasound in detecting structural defects [12].

However, a standardized first-trimester anatomic protocol should not only describe the anatomic structures but also have clearly specified standard sectional views [13]. Standardized ultrasound protocols have been used for a long period of time for performing a routine second-trimester morphological scan. Previous studies [11,12] have not clearly specified the standard sectional views needed for an appropriate FT ultrasound evaluation.

In our study, we proposed the use of actual standardized international and national protocols to increase the detection rate of fetal structural anomalies at the end of the first trimester between 11 weeks and 13 weeks + 6 days of gestation. Since the legal regulation for the termination of pregnancy is different depending on the country, we proposed to discuss the juridical impact of these anomalies at the time of detection and the implications related to the decisions that can be taken according to the Romanian laws.

## 2. Materials and Methods

We conducted an observational study over a 1-year period in the Obstetrics and Gynecology Clinic of the Emergency Clinical County Hospital from Craiova between 1 January 2024 and 31 December 2024.

The aim of our study was to identify the impact of the FT morphological ultrasound examination in the detection of fetal congenital anomalies in a low-risk population. All ultrasound examinations were performed during the FT nuchal scan by using a standard protocol for pregnancy anomaly detection [14,15]. The study was approved by the ethics committee of the University of Medicine and Pharmacy of Craiova.

We evaluated pregnancies between 11 weeks and 13 weeks + 6 days of gestation. We used ultrasound equipment that included a GE—Voluson E8 (GE Healthcare, Zipf, Austria, Version 21.1). The patients were examined using transabdominal and transvaginal approaches.

The FT morphological protocol used in this study was approved by the Romanian Society of Obstetrics and Gynecology. The scan was performed in 2D and a color Doppler mode and assessed the fetal skull (choroid plexuses, thalamus, and median septum), thorax (lung areas, diaphragm, and heart), abdomen (presence of stomach and bowel, integrity of the anterior abdominal wall, presence of the bladder, and confirmation of the paravesical vessels), and upper and lower limbs (evidence of trisegmented limbs and assessment of the fingers and toes); an assessment of the resistivity index of both uterine arteries was also performed.

**Figure 1:**
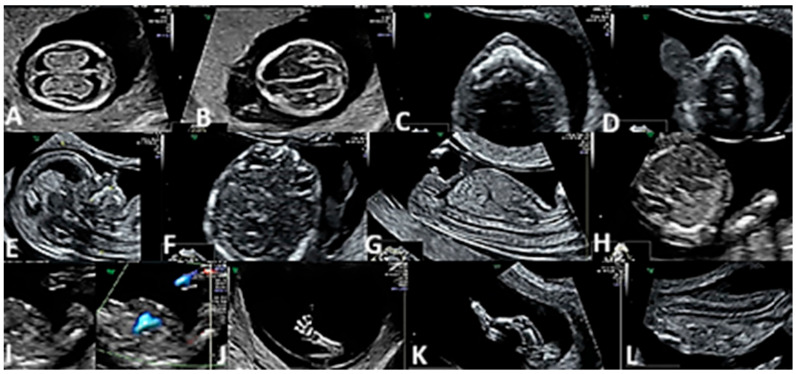
FT ultrasound morphological protocol. (**A**) Fetal brain (choroid plexus). (**B**) Fetal brain (thalamus and median septum). (**C**) Fetal face (upper lip and anterior palate). (**D**) Fetal face (lower lip and mandible). (**E**) Face sagittal view. (**F**) Fetal face (orbits and lens). (**G**) Fetal thorax and abdomen. (**H**) Fetal heart (4CV). (**I**) Fetal heart (3VV in Gray Scale and Color Doppler Mode). (**J**,**K**) Fetal limbs (upper and lower limbs). (**L**) Fetal spine.

We started the examination by assessing the situs and cardiac axis. We continued with the four-chamber view (4CV) assessed in grayscale and the color Doppler mode. The outflow tracts were assessed in the color Doppler mode. The heart examination ended with the 3-vessel view (3VV) color Doppler mode that demonstrated the confluence of the two arterial arches on the left side of the fetal spine (Figure 1).

After performing the ultrasound, the patients were counseled about the genetic screening method by maternal serum determination of PAAP-A and HCG or by the NIPT method.

In cases where the genetic screening method showed an increased risk, we performed a chorionic villous sampling (CVS). In some cases, when diagnosed with genetic disorders, the patients decided to go through with the termination of pregnancy (TOP) before the end of the first trimester.

**Figure 2:**
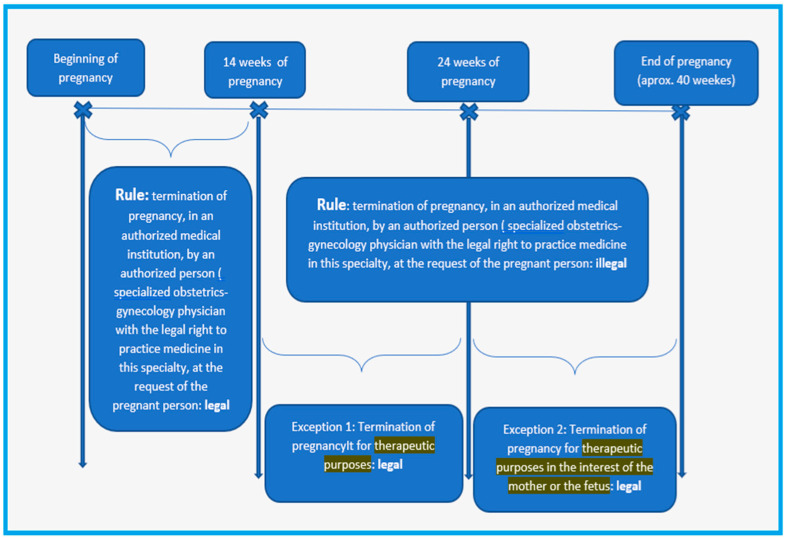
The regulation of the TOP according to the Romanian Criminal Code.

In our research, the medical law implications were related to the cases where the ultrasound was performed at about 13 weeks of gestation, and the results of the genetic screening, which arose during the FT, showed an increased pregnancy risk. In these cases, we performed amniocentesis at about 16–17 weeks of gestation, and especially, the NIPT-positive cases were confirmed by karyotyping. Still, at this gestational age of diagnosis, the Romanian law would not allow the voluntary termination of pregnancy (abortion on demand). After 14 weeks of gestation, abortion on demand becomes illegal. Termination of pregnancy could be practiced only in situations where the severity of the anomalies represents a therapeutic reason or puts the mother’s life in danger (Figure 2).

### 2.1. Patient Inclusion and Exclusion Criteria

All the patients included in the study were singletons pregnancies which had a dating scan in the first trimester. Pregnancies over 14 weeks of gestation or pregnancies ended by abortion before the NT scan were excluded from the study.

### 2.2. Statistical Analysis

Statistical analysis was performed by using a database built in Microsoft Excel Version 1520 (Microsoft Corporation, Redmond, WA, USA) and IBM SPSS Statistics 26.0 (IBM Corporation, Armonk, NY, USA). Abnormal cases are reported in tables as produced by either MS Excel or SPSS.

## 3. Results

Between 1 January 2024 and 31 December 2024, 854 pregnancies with gestational ages between 11 weeks and 13 weeks + 6 days were morphologically evaluated during the nuchal scan in the Obstetrics and Gynecology Department of the Emergency County Hospital from Craiova. In most cases (94%), we were able to complete the morphological fetal protocol by transabdominal ultrasound. In only 6% of the cases, a transvaginal ultrasound was needed due to maternal obesity, anterior fibroids, or persistent unfavorable fetal position.

Thirty-four cases resulted in miscarriage or fetal death by the time of the NT scan and were excluded from the study. Of the remaining 820 cases, 58 cases had at least one structural anomaly. In our study on a low-risk population, the incidence of fetal structural malformations was about 7% (58/820). We had an overall detection rate for the FT morphological ultrasound of 72.6% (*p* value < 0.01).

We detected 67.2% (*p* value < 0.01) of nervous system defects, 46.8% of facial anomalies, 96.9% (*p* value < 0.01) of abdominal wall defects, 37.8% (*p* value < 0.01) of limb and skeletal anomalies, and 52.2% (*p* value < 0.01) of cardiac defects.

Nuchal translucency (NT) was measured in all cases. We detected an increased NT > 3.5 mm in most of the cases that also had structural anomalies (43 cases—74.1%, *p* value < 0.01). Most of these cases are associated with major nervous system anomalies (holoprosencephaly), cardiac defects (univentricular heart, double outlet right ventricle, D-transposition of the great arteries, and ventricular septal defect), and abdominal wall anomalies (omphalocele) (Table 1).

We had an over 92% detection rate (*p* value < 0.01) for the major fetal anomalies such as anencephaly, holoprosencephaly, and exencephaly. Eleven cases were detected with major anomalies of the nervous structures, and in nine of these cases, the fetuses also had facial anomalies.

**Figure 3:**
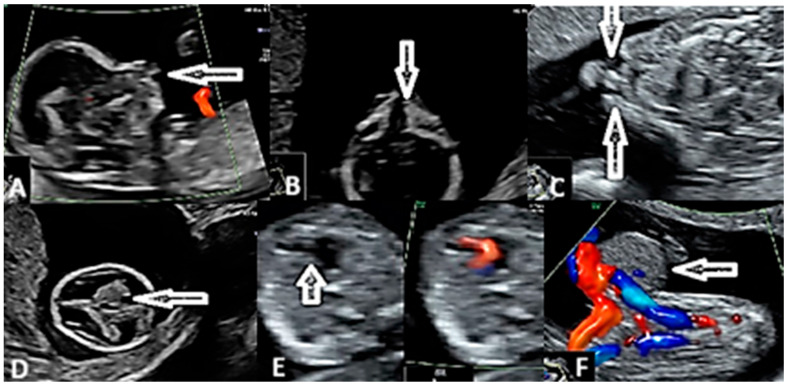
(**A**) Abnormal fetal profile. (**B**) Fetal face—cleft palate (arrow). (**C**) Fetal face—bilateral cleft upper lip (arrows). (**D**) Abnormal fetal brain. (**E**) Fetal heart—hypoplastic left heart (arrows; Left side-Grey Scale and Right side-Color Doppler Mode) (**F**) Fetal abdomen—omphalocele (arrows; Grey Scale and Color Doppler Mode).

We also had an over 95% detection rate (*p* value < 0.01) for major wall defects, such as omphalocele and gastroschisis, and major heart defects, such as atrio-ventricular septal defects and transposition of the great vessels (Figure 3).

Of the 58 fetuses detected with at least one structural abnormality, 32 (55.1%, *p* value < 0.01) cases were diagnosed with major anomalies. In these cases, the couples opted for the termination of pregnancy.

The remaining 26 cases underwent a follow-up ultrasound, and from these, 7 cases ended up in the second trimester. In these seven cases, severe fetal structural anomalies were confirmed, and the genetic diagnosis was positive. Nine cases also ended up in the late second trimester for severe fetal structural anomalies. From the rest of the remaining 10 cases, 3 cases underwent fetal intrauterine death, and 7 cases were lost to follow-up. All fetal structural anomalies are summarized in Table 2.

Our study results also have some limitations regarding the ongoing development of the fetal structures in the second and third trimesters. Some brain anomalies could not be detected because of an incomplete formation of the fetal brain in the FT. Still, some cardiac defects, such as membranous septal defects, cannot be obvious before the mild structural anomalies in the second trimester.

## 4. Discussion

Over the years, various studies have reported average detection rates for the first-trimester morphological fetal ultrasound of 50% [8,14]. The rates vary depending on the population groups, the inclusion criteria, and the different protocol standards.

A systematic review showed that the sensitivity for the detection of all types of fetal structural defects in low-risk unselected pregnancies ranged from 11.54% to 65.7% [8].

Colosi et al. [15] used a checklist of 10 planes for the detection of major fetal anomalies, which would allow more accurate fetal screenings. In this study, the detection rate of all mild structural anomalies could be increased to 43.1%, and it could be increased to 70.7% for the major structural anomalies [15]. Our study results report similar detection rates to those reported by Colosi.

Using standardized heart screenings, the diagnosis of congenital heart defects can be improved. Karim et al. [16] implemented an extensive fetal structural protocol to increase the detection rate of fetal cardiac defects in the first trimester. Our study recorded a 52.2% detection rate for such defects in the first trimester. Evaluation of the four-chamber view increased the detection of atrio-ventricular septal defects (AVSDs) and hypoplastic left heart syndrome (HLHS) cases. By using the color Doppler evaluation of the 3VV view, all of the tetralogy of Fallot (TOF) cases were identified. Interestingly, our findings suggest that the cardiac screening protocols utilized in the second trimester may also be suitable for the first trimester [17,18].

Liao et al. [19] detected one of two cases of syndactyly but no cases of polydactyly. In this study, two cases of polydactyly and one case of syndactyly were missed. The conclusion of this study highlighted the need for a close assessment of the hand and foot structures.

In a study conducted by Patru et al. [20], the researchers identified most of the cases with major structural limb anomalies in the first trimester and highlighted the role of the morphological ultrasound to improve pregnancy outcomes and surgical management.

Increased NT thickness above 3.5 mm has been associated with a high risk of genetic disorders that are often associated with structural anomalies [21,22,23]. In chromosomally normal fetuses, an increased NT can be a marker of other associated anomalies such as cardiac, gastrointestinal, or musculoskeletal defects, highlighting the need for a careful fetal anatomical assessment [24,25].

Our study results show that fetuses with an increased NT are more often associated with structural anomalies compared to fetuses with normal NT. Nervous system defects, congenital heart defects, and limb defects are associated with NT > 3.5 mm. Multiple defects indicate a potential necessity for an ongoing screening for structural defects.

Still, there will always be limitations for evaluating the fetal structures in the first trimester, depending on the operator’s experience, the standardized protocol used, and the further development of the fetal structures, especially for the brain and heart. Therefore, the first-trimester morphological ultrasound could not replace further follow-up during pregnancy, especially between 20 and 24 weeks of gestation. Also, beyond this gestation age, further anomalies can be detected in the third trimester.

Regarding the fetal heart, some cardiac defects like aortic stenosis, pulmonary stenosis, and Ebstein anomaly evolve in late pregnancy. Therefore, they cannot be diagnosed in the first trimester. A discrete coarctation of the aorta can be misdiagnosed due to the patency of the ductus arteriosus. Still, secundum atrial defects can be misdiagnosed due to the patency of the foramen ovale. Also, some fetal anomalies, such as abdominal wall defects, can resolve later in pregnancy, and in these cases, the couples may end up with a pregnancy with a healthy fetus.

The medical law impact of these structural and genetic fetal anomalies depends on the type of structural abnormality (major or minor) and the time of the diagnosis (first trimester or second trimester) in relation to each state’s laws.

If an FT morphological ultrasound examination is not made or is misinterpreted, this could lead to complaints or legal actions, including medical malpractice for several reasons [26].

Parents might argue that, if they had known about a fetal abnormality beforehand, they might have considered terminating the pregnancy. Additionally, the parents might claim that a lack of intrauterine treatment or immediate treatment after birth resulted in permanent damage or a disability that could have been prevented. Couples could assert that they had the right to be informed about abnormal test results during pregnancy so they could prepare for having a baby with an impairment or structural defect [27].

Obstetricians must fulfill their legal obligation to inform the patient in order for the patient to decide based on medical evidence. Only through adequate information can the patient give consent for termination of the pregnancy [28,29,30].

Facilitating safe care for the mother requires a good knowledge of laws and clinical guidelines [31]. According to the legal literature, the obstetrician–gynecologist will interrupt the course of the pregnancy in two main situations: in case of voluntary demand or by therapeutic reasons [32].

The voluntary termination of pregnancy (VTOP) is based on the patient’s request, without needing the existence of a reason, but within a period established by law. The therapeutic termination of pregnancy (TOP) is an induced abortion following a diagnosis of medical necessity. At the international level, the regulations regarding Sexual and Reproductive Health include, in the category of medical grounds that justify the necessity of terminating the pregnancy, the situations in which the mother’s life is in danger, general health, therapeutic reasons (it should be noted that some regulations refer strictly to physical health), or when there are data regarding the lack of fetal viability or the presence of anomalies [33,34].

TOP is carried out in cases of fetal unviability or to avoid the risk of substantial harm to the mother. This type of termination of pregnancy requires a clinical evaluation by a physician.

The analysis of the first situation is easy; the doctor will only analyze the mother’s capacity for discernment and the formal and substantive conditions of the medical document request. The second situation regarding the existence of therapeutic reasons or the situation in which the life of the mother or the fetus is put in danger involves many discussions.

Limited by the legal norms, the obstetrician will have to identify clinical situations that are circumscribed by therapeutic reasons and will have to prove their belonging through medical evidence. Any deviation from the legislation will attract legal liability.

Unfortunately, the lack of clarity of the legal norms does not provide a solid and efficient framework of what the therapeutic reasons are and what medical protocol should be followed [35]. Doctors generally have limited knowledge about the related legislation [36].

Moreover, health professionals must clarify all the circumstances to identify the cases where the termination of pregnancy is permitted by law. The subsequent step is counseling the patient by referring to the relevant legal norms [37].

The Romanian Criminal Code stipulates in Art. 201 (6) that “It is not a criminal offense to interrupt the course of pregnancy for therapeutic purposes by a specialist obstetrician-gynecologist up to the age of twenty-four weeks of pregnancy or to discontinue the course of pregnancy for therapeutic purposes in the interest of mother or fetus” [38]. The lack of legislative provisions that identify the therapeutic reasons through an expressed, limiting enumeration of medical conditions is likely to cause confusion.

Our study demonstrates the value of the first-trimester ultrasound in detecting fetal anomalies by using a standardized protocol. An experienced ultrasound operator is a very important factor in performing a morphological ultrasound for the detection of fetal structural anomalies in the late first trimester. The detection of fetal structural defects between 11 and 13 weeks of gestation can be improved due to the other associated structural or genetic markers. Also, the detection rate can be improved using both transabdominal and transvaginal ultrasound approaches [39,40]. Perinatal autopsy at this gestational age is very difficult, but it is possible using a three-dimensional (3D) reconstruction of the histological sections of the fetal heart [41].

## 5. Conclusions

Due to the evolving fetal structures during pregnancy and the risk associated with the developing diseases, together with the limitations of the fetal structural assessment in the first trimester, practitioners should re-evaluate the fetal structures in the second and third trimesters of pregnancy. Our study results also confirmed these data and the fact that this primary evaluation should not replace further evaluation. Our study also demonstrated that at the time of the NT measurement, we identified most of the fetal structural anomalies and genetic disorders. We had an over 92% detection rate (*p* value < 0.01) for the major fetal anomalies.

By using the recommendations of a standardized screening protocol, we can increase the detection rate of fetal structural anomalies. Although some fetal structural anomalies can be misdiagnosed in the first trimester, major structural anomalies such as cardiac and nervous system anomalies can be diagnosed.

At the end of the FT, the increased NT values indicate the need for a detailed structural fetal assessment. The use of a standardized first-trimester morphological protocol offers new options, including genetic testing, couples counseling, prognostic information, and appropriate management. An ongoing screening in the second and third trimesters is mandatory because other fetal structures develop later in pregnancy. Therefore, an early genetic and structural diagnosis using an extended FT morphological protocol and invasive intrauterine interventions (chorionic villous sampling) would improve the pregnancy outcome due to more accurate couples counseling, which would lead to earlier decisions to terminate the pregnancy.

When a fetal anomaly is diagnosed, pregnant women have the right to be informed about all the risks, medical interventions, and existing alternatives to the proposed procedures, including non-treatment and non-compliance with medical recommendations. Depending on the severity of the anomalies and the pregnancy period, the legality of the pregnancy termination will be evaluated. Therefore, the identification of fetal anomalies and medical and legal counseling can become the main determinants in terminating a pregnancy.

## Figures and Tables

**Table 1:** Most common fetal anomalies associated with an increased NT > 3.5 mm.

Anomaly	Number of Cases		Percent	*p* Value
Nuchal translucency (NT)	NT < 3.5 mm—15	NT > 3.5 mm—43	43/58 (74.1%)	<0.01
Holoprosencephaly	4	4	4/58 (6.9%)	<0.01
Double outlet right ventricle	0	2	2/58 (3.4%)	<0.01
D-transposition of the great arteries	0	2	2/58 (3.4%)	<0.01
Ventricular septal defect	0	1	1/58 (1.7%)	<0.01
Omphalocele	3	6	6/58 (10.3%)	<0.01

**Table 2:** Fetal structural anomalies.

Fetal Structural Anomalies First Trimester	Cases	Percentage	*p* Value
Nervous system	23	23/58 (39.6%)	<0.01
Holoprosencephaly	11	11/58 (18.9%)	
Anencephaly	7	7/58 (12%)	<0.01
Exencephaly	5	5/58 (8.62%)	<0.01
Face	7	7/58 (12%)	
Cleft lip only	4	4/58 (6.89%)	<0.01
Cleft palate only	2	2/58 (3.4%)	
Cleft lip and palate	1	1/58 (1.7%)	
Thorax and abdomen	12	12/58 (20.6%)	
Omphalocele	9	9/58 (15.5%)	
Gastroschisis	3	3/58 (5.17%)	
Heart	16	16/58 (27.5%)	
Double outlet right ventricle	2	2/58 (3.4%)	<0.01
D-transposition of the great arteries	2	2/58 (3.4%)	<0.01
Univentricular heart	4	4/58 (6.8%)	<0.01
Complex cardiac malformation	5	5/58 (8.62%)	<0.01
Tricuspid valve atresia with VSD	1	1/58 (1.7%)	<0.01
Major interventricular septal defect (VSD)	1	1/58 (1.7%)	<0.01
Tetralogy of Fallot	1	1/58 (1.7%)	<0.01
Limbs	2	2/58 (3.4%)	
Club foot	1	1/58 (1.7%)	
Polydactyly	1	1/58 (1.7%)	
TOTAL	58	100%	<0.01

## Data Availability

All data presented here are available from the authors upon reasonable request.
